# Comparison of Satisfaction With Comorbid Depression Care Models Among Low-Income Patients With Diabetes

**DOI:** 10.1177/2374373519884177

**Published:** 2019-10-31

**Authors:** Olivia Evanson, Shinyi Wu

**Affiliations:** 1 Daniel J. Epstein Department of Industrial and Systems Engineering, 5116 University of Southern California, Los Angeles, CA, USA; 2 Suzanne Dworak-Peck School of Social Work, 5116 University of Southern California, Los Angeles, CA, USA; 3 Edward R. Roybal Institute on Aging, 5116 University of Southern California, Los Angeles, CA, USA

**Keywords:** patient-reported outcomes, patient satisfaction, depression, telecommunication technology, care-management, type 2 diabetes, safety-net care, comparative effectiveness

## Abstract

**Introduction:**

Patient satisfaction is a patient-reported outcome with the potential to assess and improve the quality of newer care-management models such as remote patient monitoring using telecommunication technology.

**Objective:**

To evaluate differences in patient satisfaction among 3 care management groups in a comparative effectiveness trial.

**Methods:**

This study analyzed a comparative effectiveness trial that tested automated remote assessment technology–facilitated comorbid depression care-management (TC, n = 254) in comparison to team-supported depression care (SC, n = 228) and usual primary care (UC, n = 218) among low-income patients with type 2 diabetes. Relationships between patient satisfaction and care group were evaluated at each 6-month phase up to 18 months using linear regression models that controlled for depression status, diabetes symptoms, patient characteristics, and study group differences.

**Results:**

While receiving care management, SC and TC patients were significantly more satisfied with depression care than UC patients. No consistently significant associations between patient satisfaction and patient characteristics or disease symptoms were found.

**Conclusions:**

Patient satisfaction was found to be influenced by elements of care-management, not by patient characteristics or disease symptoms. Results suggest greater patient satisfaction with depression care in a care-management model than UC, whether through clinician team support or automated remote monitoring technology.

## Introduction

With increasing technological capabilities for remote symptom assessment, monitoring, and patient–provider communication, ambulatory care settings have begun embracing newer care models outside traditional face-to-face care to improve care-management (1,2). Many studies have demonstrated the effectiveness of remote patient care-management using telecommunication technology to increase access to care while decreasing health-care costs (3–9). Furthermore, measuring patient satisfaction provides the patients’ perspectives and experience of health-care quality in various care management models (10–12), and analyzing patient satisfaction with care-management provides an opportunity to understand how patient satisfaction is influenced by alternative models of care (13,14). Recently, studies have successfully evaluated patient satisfaction, postintervention in pre–post studies, as an assessment of the care received through remote care-management interventions (15,16). However, we currently need greater knowledge on how patient satisfaction is affected by variations in care-management models and what this tells us about the quality of care experience provided.

The Diabetes and Depression Care-Management Trial (DCAT) provides an opportunity to compare 2 care management models to usual care (UC). The DCAT was a multisite, quasi-experimental trial with 3 trial groups of low-income, primarily Latino patients with type 2 diabetes from the Los Angeles County Department of Health Services (LAC-DHS) (17). The trial assigned 6 provider teams and their 1400 patients seen in 8 LAC-DHS ambulatory care clinics to 1 of 3 comorbid depression care models:

1. Usual care: Patients’ primary care team to provide usual diabetes and depression care.
2. Supported care (SC): Patients with poor diabetes control were placed in a protocol-driven, nurse-led diabetes disease management program (DMP) for a limited time (typically 6-9 months) to improve their diabetes knowledge, self-management, treatment, and follow-up. The DMP team included physicians, nurse practitioners, and social workers supported by a web-based disease management registry (DMR) for care management. During DCAT, DMP clinical social workers performed depression screening and symptom monitoring as well as problem-solving therapy, while DMP nurse practitioners provided antidepressant treatment following a depression treatment protocol.
3. Technology-facilitated care (TC): The same as SC, plus a care approach using an automated telephonic assessment (ATA) system for periodic patient remote monitoring. The ATA system performed depression screening, symptom monitoring, and assessment of important self-care activities (ie, physical activity, medication adherence, and/or practicing problem-solving) once every month for depressed patients and every 3 months for nondepressed patients at a patient-preferred call time. In addition, the DMR was enhanced by computer algorithms to notify and task different DMP team members to provide collaborative depression care (18).

Compared to UC, the ATA technology and nurse/social worker–delivered DMP had the potential to affect how patients perceive care available to them through increased frequency in health assessment and likelihood of contact with and support by providers. With the increased frequency of assessment and access to providers, we hypothesized that, compared to UC, both TC and SC would increase patient satisfaction with the care available for emotional problems and diabetes symptoms.

The Institute for Healthcare Improvement’s triple aim initiative identifies 3 aims for health-care interventions: improved health outcomes, reduced costs, and improved patient experience. Previous DCAT analyses have demonstrated improved health outcomes and lowered costs (19,20) and have investigated patient experience through patient acceptance of the technology (21,22). In this research, our goal is to analyze comparative patient experience over 3 phases lasting 18 months, where every 6 months patients were exposed to different elements of care-management due to the DMP design and ATA intervention. Using the data collected from DCAT, this analysis answers the following research question: What is the difference in patient satisfaction when patients were exposed to different ambulatory care-management models over time, after controlling for patient differences?

The significance of this research is to understand whether patient satisfaction is sensitive to elements of care-management or is biased by personal characteristics, depression status, or diabetes symptoms (10). This study will help understand the value of using patient satisfaction to assess and improve quality of care experience with newer care-management models, such as comorbid disease management in the context of population health.

## Methods

### Study Design and Data Source

**Figure 1.**
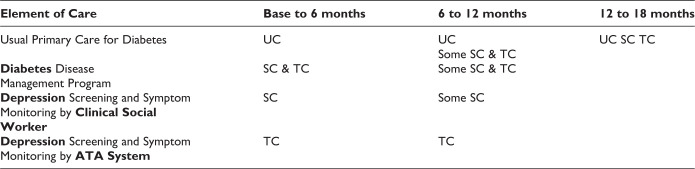
Varying care management models over time in Diabetes-Depression Care Management Trial (DCAT)’s 3 study groups.

The study design was reviewed and approved by the institutional review board (IRB) of USC Health Sciences, LA Biomedical Research Institute IRB, and the Olive View–UCLA Education and Research Institute IRB. Participants provided written informed consent. Participant inclusion criteria included the following: patients were 18 years or older, had a working phone number, spoke English or Spanish, and could read and understand the consent form. Exclusion criteria included the following: patients with baseline possible suicide ideation (Patient Health Questionnaire [PHQ]-9, item 9 response in more than half the days to nearly every day), cognitive impairment (Short Portable Mental Health Status Questionnaire scores of <5) (23), alcohol abuse (2 or more CAGE items from the quantity–frequency index, and questions about the patient’s perception of substance use) (24), or recent lithium/antipsychotic medication use. Patients were assigned to 1 of the 3 study groups based on the clinic from which they were recruited, thus utilizing a quasi-experimental comparative effectiveness design. Figure 1 depicts the varying care-management elements over time in the 3 study groups. Patients were enrolled in DCAT for 18 months. Data were collected at 6-month intervals; baseline was in-person at the study sites, and the 3 follow-ups were telephone interviews with a Spanish–English bilingual, group-blinded interviewer. Only a subset of the DCAT participants, that is, 700 participants who completed 4 waves of the interview data from baseline to 18 months, were included in the analyses.Measures

There were 2 outcomes of interest: (1) How satisfied are you with the clinical help you received with emotional problems and (2) How satisfied are you with the overall health care available to you for your diabetes? The outcomes were recorded on a 5-point Likert-type scale where 1 indicated “very dissatisfied” and 5 indicated “very satisfied.” Measures of patient demographics and baseline clinical characteristics are listed in Results section (Table 1).

**Table 1.** Baseline Patient Characteristics.

Variable	UC, n = 218	SC, n = 228	TC, n = 254	*P*
Age, mean (SD)	55.78 (8.76)	51.63 (9.69)	52.28 (8.80)	<.0001
Gender (male = 1), n (%)	66 (30.28)	85 (37.28)	87 (34.25)	.2940
Pref. language Spanish, n (%)	196 (89.91)	20 (87.72)	237 (93.31)	.4038
Education (<high school), n (%)	178 (82.03)	157 (68.86)	187 (73.62)	.0054
Marital status (married = 1), n (%)	128 (58.72)	144 (63.16)	120 (47.24)	.0013
Financial stresses, mean (SD)	4.32 (2.50)	3.78 (1.87)	4.30 (2.10)	.0105
Depression status,^a^ n (%)	70 (31.11)	83 (36.40)	61 (24.02)	.0109
Diabetes symptoms,^b^ mean (SD)	1.72 (.64)	1.81 (.68)	1.56 (.52)	<.0001
Satisfaction with Depression Care, mean (SD)	4.18 (1.04)	4.75 (.56)	4.57 (.63)	<.0001
Satisfaction with Diabetes Care, mean (SD)	4.57 (.82)	4.86 (.37)	4.68 (.54)	<.0001

Abbreviations: SC, supported care; SD, standard deviation; TC, technology-facilitated care; UC, usual care.

^a^ Assessed by the 9-item Patient Health Questionnaire. Scores ranged from 0 to 27; scores greater than 9 indicate depression (25).

^b^ Assessed by the Whitty 9-Item Diabetes Symptoms Scale. Item scores ranged from 1 to 5; higher scores indicate more severe diabetes symptoms (26).

The primary independent variable was care-management group. Two of the 3 groups (SC and TC) were included as intervention groups, and UC was the control group. There were many additional control variables in this analysis. The first was depression status, measured using the PHQ-9, which scores each of the 9 *Diagnostic and Statistical Manual of Mental Disorders* (Fourth Edition) criteria as “0” (not at all) to “3” (nearly every day); a cumulative score >9 indicates having depression (25). The second additional control variable was diabetes symptoms, which was measured with the Whitty-9 Diabetes Symptom Scale, a 9-item questionnaire including abnormal thirst, blurred vision, urinated a lot of water during the day, felt unusually hungry, felt shaky, had cold hand and feet, felt very sleepy during the day, had feeling of pins and needles, and felt faint or fainted, where mean scores indicate the frequency of symptom experience (26). These 2 variables were included to adjust for patient disease status. A collection of patient characteristics was included to adjust for patient differences as well as to provide insight into any patient satisfaction differences by characteristics in this study population (27). These variables included age, gender, preferred language spoken (English or Spanish), education level (less than high school or more than high school), marital status (married or unmarried), and financial stresses (12-item score measuring financial difficulties). In addition, propensity scores, as developed and used in a previous DCAT analysis (20), were included to adjust for the quasi-experimental design so that differences in the care-management model could be observed above patient differences by group.

### Analysis

Linear regression models were used to estimate comparative treatment effects. Dependent variables in all regression analysis were the 6-, 12-, and 18-month satisfaction scores. Previous research has shown that modeling Likert-type scale items as continuous variables, even when normality assumptions are violated, provide a correct result (28). Thus, the satisfaction items were modeled as continuous variables for this analysis although slight violations of normality were present.

The satisfaction items were individually regressed on the independent variable, care-management group, and all control variables: current period depression status, current period diabetes symptoms, previous period satisfaction score, age, gender, language spoken, education level, marital status, financial stresses, and propensity score.

After running the linear regression models, global Wald tests were used to test for significant differences in satisfaction between the care-management groups at all study periods (6, 12, and 18 months). All statistical analyses were conducted at 0.05 significance level (2 tailed) using SAS 9.4 and Stata 15.

It should be mentioned that repeated measures analysis of variance (ANOVA) may appear to be a good fit for analyzing these data; however, this was a comparative effectiveness trial, where the intervention was changing at each phase. Thus, repeated measures ANOVA would not provide the same insight into the effects of the changing intervention at each phase that the selected methods provide. Additionally, we checked clinic and care team significance against care-management group, and results showed care-management group was consistently the most significant predictor of satisfaction.

## Results

### Sample Characteristics

**Figure 2.**
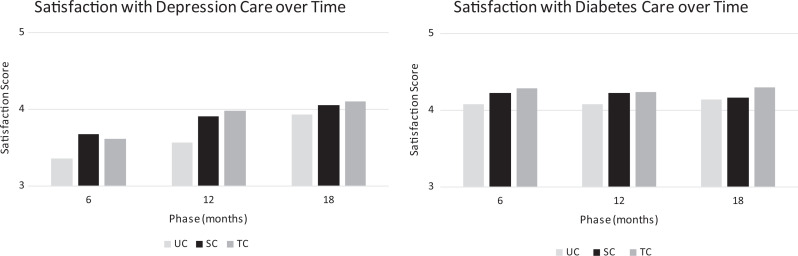
Unadjusted mean satisfaction score with (A) depression care and (B) diabetes care.

Table 1 provides sample size and characteristics of patients in the 3 DCAT groups. The study population included primarily low-income Latinos in the Los Angeles area. Most patients were women, and approximately one-third of patients in each study group were depressed. Figure 2 provides unadjusted mean satisfaction scores over time.

### Regression Analysis Results

Wald tests of study group significance identified significant differences in satisfaction among the care management groups in the linear regression models as shown in Table 2. The test identified significant relationships between 6- and 12-month satisfaction with depression care and study group (*P* = .0113, *P* < .0001). Controlling for all other covariates, patients in TC and SC both were significantly more satisfied than UC patients with depression care at 6- and 12 months (*P* = .0037, *P* < .0280 and *P* < .0001, *P* < .0001 respectively). The levels of satisfaction in the TC and SC were not statistically different. The Wald test did not identify any significant relationships between satisfaction with diabetes care and study group at any phase.

**Table 2.** Wald Test *P* Values of Satisfaction Differences Between Care Management Groups.

Test	Satisfaction With Depression Care			Satisfaction With Diabetes Care		
	6 Months	12 Months	18 Months	6 Months	12 Months	18 Months
Group UC = SC = TC	.0113	<.0001	.2314	.2700	.2019	.2777
Group UC = SC	.0037	<.0001	.0909	.1211	.0738	.9253
Group UC = TC	.0280	<.0001	.2507	.2214	.3239	.1947
Group SC = TC	.3472	.9611	.4813	.6534	.3443	.1667

Abbreviations: SC, supported care; TC, technology-facilitated care; UC, usual care.

Other noteworthy results include nonsignificant negative associations between satisfaction with depression care and PHQ-9 depression status, with 18-month follow-up being an exception in reaching *P* < .05. There were also negative associations between satisfaction with diabetes care and Whitty-9 Diabetes Symptom Scale, but the association was only statistically significant at 6-month follow-up. No patient characteristics consistently displayed associations with satisfaction with depression care. The only patient characteristic to consistently display a moderate association with satisfaction with diabetes care was age at 12 and 18 months. Appendices 1 and 2 provide the complete regression output.

In a post ad hoc analysis, 3 additional satisfaction items measured in DCAT were examined: satisfaction with clinical scheduling services, satisfaction with respect from providers, and satisfaction with medical decision-making in care received (results available upon request). Some significant relationships were found between the service-oriented satisfaction items and the care-management models but not with disease symptoms or patient characteristics.

## Discussion

### Principal Findings

Patient satisfaction with depression care was found to be higher in care-management intervention groups, and satisfaction with diabetes care was found to have no differences between the groups. No consistently significant associations between patient satisfaction and disease symptoms or patient characteristics were found. These findings show that patient satisfaction is responsive to the care processes influenced by the interventions as well as the other aspects of care (aside from the intervention) that changed during the study period, without heavy affect by patient characteristics or disease symptoms.

Patient satisfaction measures 3 intertwined components of the satisfaction construct: expectations, value, and occurrences (29). Patient satisfaction measures are affected more by actual occurrence than perceived-to-be occurrence of interaction with providers; the intervention improving attributes of care that patients value highly; and more health-care interactions taking place, giving patients more opportunities to interact with the care that provides higher value. Because the DMP providers and social workers were trained to practice the depression care protocol (17), patients were prone to be more satisfied with the emotional care they received in the DMP. Patients did not sustain the higher level of satisfaction, as they transitioned back to usual primary care. The ATA technology prompted automated patient depression monitoring in the TC group, monthly for depressed patients and quarterly for nondepressed patients. Although reducing provider workload in monitoring the entire patient population compared to the SC, the ATA system in TC was able to identify patients in need of care and tasked providers to reach those patients. Albeit low frequency, the increased contact with providers facilitated by the ATA was when care was needed. It seemed to increase satisfaction with emotional care for patients in the TC group equally as the SC group.

### Comparison to Literature

A 12-month study of patients with type 2 diabetes and comorbid chronic diseases, including depression, was conducted with 2 study arms, UC and clinician-SC. Their study population included primarily white older adults from Washington state; they were not specified as low income. The researchers found patients in the SC group were significantly more satisfied with depression care than UC at 6 months (87% vs 62%) and at 12 months (90% vs 55%) (30). Consistent with those results, we also found that patients with clinician-supported depression care were significantly more satisfied with depression care at 6 months (58% vs 40%) and 12 months (78% vs 56%). Our analysis additionally investigated the technology-facilitated assessment and found that patients receiving ATA depression support was also significantly more satisfied with depression care available to them at 6 months (52% vs 40%) and 12 months (80% vs 56%), providing support that this newer care-management approach can equally enhance patient satisfaction with care. The combination of significant study population differences and similar results between our 2 analyses provides generalizability of the findings of higher satisfaction with depression care in a SC model, whether through clinician support or ATA technology.

A different study had a study population most similar to our study and used technology-facilitated assessment and follow-up support system for chronic disease; this study also found high satisfaction rates with the telephone calls for SC (31). Although the study population and intervention were similar, the study assessed satisfaction with the technology but not the satisfaction with care.

Not only has higher patient satisfaction with technology been found in previous DCAT analysis (22), our study evaluated satisfaction with the care available through the intervention models and found higher satisfaction rates in both the SC and the TC models. In addition, where the literature shows mixed results of differences in satisfaction by patient characteristics (27), our study provides further empirical evidence of limited effects of patient characteristics on patient satisfaction with care received.

### Limitations and Future Work

The first limitation of this analysis is the quasi-experimental trial design. This was addressed by including generalized propensity scores for each patient to account for differences in patient characteristics between the study groups.

Second, attrition of 706 (50%) patients over the 18-month study period was a substantial reduction in the study sample size. However, there were no significant differences between the patients who completed the study and those who dropped out aside from by language spoken, gender, and education level. English-speaking, educated males were more likely to drop out. This result could have biased the satisfaction levels for the remaining sample. Patients who remained in the study could have exhibited lower levels of satisfaction due to limited knowledge on how to advocate for themselves, as this association has previously been found (32).

A third limitation was the duration of time since patients last received care and the study interview time. Although we see improvements in satisfaction at 6 months and 18 months, there was a dip in satisfaction between baseline and 6 months. A negative correlation between length of time since last visit and patient satisfaction was found (satisfaction with depression care ρ = −.024 and satisfaction with diabetes care ρ = −.078). Baseline assessment was done in-person immediately after patients had an appointment in a study clinic, while the follow-up assessment was done as a telephone interview that did not coincide with the patient’s last medical appointment. In the meantime, the patient may have received care from medical practitioners who were not included in the study. Thus, the measurement of patient satisfaction with care might be biased by memory recall issue, lack of access to care, or other care services than those intended for the study. Future analyses of patient satisfaction can investigate the occurrence of the same phenomenon.

## Conclusions

This study evaluated patient satisfaction with diabetes and depression comorbid care-management and ATA technology, controlling for patient characteristics and disease symptoms. The analysis showed that patient satisfaction is influenced by aspects of care provided by the intervention and not by patient characteristics or disease symptoms. Care-management models increased patient satisfaction with depression care compared to UC, whether through clinician support or through automated remote monitoring technology.

## Supplemental Material

Appendix_1 - Comparison of Satisfaction With Comorbid Depression Care Models Among Low-Income Patients With Diabetes Click here for additional data file. Appendix_1 for Comparison of Satisfaction With Comorbid Depression Care Models Among Low-Income Patients With Diabetes by Olivia Evanson and Shinyi Wu in Journal of Patient Experience

Appendix_2 - Comparison of Satisfaction With Comorbid Depression Care Models Among Low-Income Patients With Diabetes Click here for additional data file. Appendix_2 for Comparison of Satisfaction With Comorbid Depression Care Models Among Low-Income Patients With Diabetes by Olivia Evanson and Shinyi Wu in Journal of Patient Experience

## Appendix

**Table A1.** Satisfaction With Depression Care Regression Output.

Phase	6 Months		12 Months		18 Months	
	Coefficient	*P*	Coefficient	*P*	Coefficient	*P*
SC group	0.30	.004	0.39	<.001	0.14	091
TC group	0.21	.028	0.39	<.001	0.09	.251
SC propensity score	0.11	.574	0.04	.810	−0.04	.790
TC propensity score	0.02	.911	0.31	.117	−0.08	.637
Previous phase satisfaction	0.10	.031	0.11	.001	0.18	<.001
Age	0.01	.088	0.004	.240	0.01	.032
Gender (male = 1)	−0.003	.967	0.04	.535	0.06	.325
Preferred language (Spanish = 1)	0.06	.589	−0.11	.330	−0.04	.713
Education (<high school)	0.01	.949	0.10	.209	0.07	.355
Marriage status (1 = married)	−0.05	.508	−0.005	.945	0.02	.666
Economic status	−0.08	<.0001	−0.004	.755	0.001	.952
Indicator PHQ-9 > 9 (1 = depressed)	−0.09	.375	−0.12	.172	−0.26	.002
Whitty-9 Diabetes Symptom Scale	−0.03	.648	−0.05	.371	0.01	.852
Constant	2.90	<.001	2.99	<.001	2.65	<.001

**Table A2.** Satisfaction With Diabetes Care Regression Output.

Phase	6 Months		12 Months		18 Months	
	Coef.	*P*	Coef.	*P*	Coef.	*P*
SC group	0.15	.121	0.14	.074	−0.01	.925
TC group	0.11	.221	0.07	.324	0.10	.195
SC propensity score	1.14	.433	0.08	.601	0.19	.204
TC propensity score	0.18	.376	0.28	.098	−0.10	.566
Previous phase satisfaction	0.08	.140	0.18	<.001	0.26	<.001
Age	0.004	.234	0.008	.010	0.01	.025
Gender (male = 1)	0.10	0.159	−0.03	.621	0.04	.461
Preferred language (Spanish = 1)	−0.07	.515	0.05	.601	0.06	.534
Education (<high school)	0.04	.614	0.05	.513	0.03	.716
Marriage status (1 = married)	−0.21	.002	0.04	.526	−0.08	.144
Economic status	−0.05	.001	−0.02	.117	−0.001	.942
Indicator PHQ-9 > 9 (1 = depressed)	−0.02	.822	−0.01	.845	−0.07	.392
Whitty-9 Diabetes Symptom Scale	−0.12	0.043	−0.03	.563	−0.04	.458
Constant	4.05	<.001	2.96	<.001	2.75	<.001
