# Supplementary material for: Comparison of Satisfaction With Comorbid Depression Care Models Among Low-Income Patients With Diabetes
Source: J Patient Exp. 2019 Oct 31;7(5):734–41. doi: 10.1177/2374373519884177 (PMC7705841; doi:10.1177/2374373519884177)
Supplement: Appendix_1 - Comparison of Satisfaction With Comorbid Depression Care Models Among Low-Income Patients With Diabetes [file Appendix_1.docx]

Appendix 1. Satisfaction with Depression Care Regression Output

| **Phase** | **6-months** | | **12-months** | | **18-months** | |
| --- | --- | --- | --- | --- | --- | --- |
|  | **Coef.** | **p** | **Coef.** | **p** | **Coef.** | **p** |
| SC Group | 0.30 | .004 | 0.39 | <.001 | 0.14 | .091 |
| TC Group | 0.21 | .028 | 0.39 | <.001 | 0.09 | .251 |
| SC Propensity Score | 0.11 | .574 | 0.04 | .810 | -0.04 | .790 |
| TC Propensity Score | 0.02 | .911 | 0.31 | .117 | -0.08 | .637 |
| Previous Phase Satisfaction | 0.10 | .031 | 0.11 | .001 | 0.18 | <.001 |
| Age | 0.01 | .088 | 0.004 | .240 | 0.01 | .032 |
| Gender (male=1) | -0.003 | .967 | 0.04 | .535 | 0.06 | .325 |
| Preferred Language (Spanish=1) | 0.06 | .589 | -0.11 | .330 | -0.04 | .713 |
| Education (< high school) | 0.01 | .949 | 0.10 | .209 | 0.07 | .355 |
| Marriage Status (1=married) | -0.05 | .508 | -0.005 | .945 | 0.02 | .666 |
| Economic Status | -0.08 | <.0001 | -0.004 | .755 | 0.001 | .952 |
| Indicator PHQ-9 > 9 (1=depressed) | -0.09 | .375 | -0.12 | .172 | -0.26 | .002 |
| Whitty-9 Diabetes Symptom Scale | -0.03 | .648 | -0.05 | .371 | 0.01 | .852 |
| Constant | 2.90 | <.001 | 2.99 | <.001 | 2.65 | <.001 |
