# Supplementary material for: Comparison of Satisfaction With Comorbid Depression Care Models Among Low-Income Patients With Diabetes
Source: J Patient Exp. 2019 Oct 31;7(5):734–41. doi: 10.1177/2374373519884177 (PMC7705841; doi:10.1177/2374373519884177)
Supplement: Appendix_2 - Comparison of Satisfaction With Comorbid Depression Care Models Among Low-Income Patients With Diabetes [file Appendix_2.docx]

Appendix 2. Satisfaction with Diabetes Care Regression Output

| **Phase** | **6-months** | | **12-months** | | **18-months** | |
| --- | --- | --- | --- | --- | --- | --- |
|  | **Coef.** | **p** | **Coef.** | **p** | **Coef.** | **p** |
| SC Group | 0.15 | .121 | 0.14 | .074 | -0.01 | .925 |
| TC Group | 0.11 | .221 | 0.07 | .324 | 0.10 | .195 |
| SC Propensity Score | 1.14 | .433 | 0.08 | .601 | 0.19 | .204 |
| TC Propensity Score | 0.18 | .376 | 0.28 | .098 | -0.10 | .566 |
| Previous Phase Satisfaction | 0.08 | .140 | 0.18 | <.001 | 0.26 | <.001 |
| Age | 0.004 | .234 | 0.008 | .010 | 0.01 | .025 |
| Gender (male=1) | 0.10 | 0.159 | -0.03 | .621 | 0.04 | .461 |
| Preferred Language (Spanish=1) | -0.07 | .515 | 0.05 | .601 | 0.06 | .534 |
| Education (< high school) | 0.04 | .614 | 0.05 | .513 | 0.03 | .716 |
| Marriage Status (1=married) | -0.21 | .002 | 0.04 | .526 | -0.08 | .144 |
| Economic Status | -0.05 | .001 | -0.02 | .117 | -0.001 | .942 |
| Indicator PHQ-9 > 9 (1=depressed) | -0.02 | .822 | -0.01 | .845 | -0.07 | .392 |
| Whitty-9 Diabetes Symptom Scale | -0.12 | 0.043 | -0.03 | .563 | -0.04 | .458 |
| Constant | 4.05 | <.001 | 2.96 | <.001 | 2.75 | <.001 |
